# INCREASING THE SENSE OF IDENTITY AND INTEGRITY: AN INTERVENTION STUDY OF ACTIVE AGING AND AUTOBIOGRAPHY WORKSHOPS

**DOI:** 10.1093/geroni/igac059.2401

**Published:** 2022-12-20

**Authors:** Tomoko Ikeuchi, Mayuko Ono, Sachiko Yamazaki, Cullen Hayashida, Michiyo Tomioka, Chiho Shimada, Hisao Osada

**Affiliations:** Tokyo Metropolitan Institute of Gerontology, Tokyo, Tokyo, Japan; Tokyo Metropolitan Institute of Gerontology, Tokyo, Tokyo, Japan; Bunkyo Gakuin University, Fujimino, Saitama, Japan; University of Hawaii, Honolulu, Hawaii, United States; University of Hawaii, Honolulu, Hawaii, United States; Saku University, Saku, Nagano, Japan; J.F. Oberlin University, Machida, Tokyo, Japan

## Abstract

Identity and integrity (i.e., a sense of coherence and wholeness) are important elements for psychosocial development in later stages of life as described in Erikson’s life-span developmental theory. This study aimed to develop an intervention program to increase the sense of identity and integrity among older adults. The program included two types of workshops to examine the combined impact of searching for one’s post-retirement identity and clarifying personal goals and purpose for later life. The first workshop, Introductory of Active Aging, focused on providing the knowledge and skills necessary to prepare for life after retirement. The second workshop, Creating Your Life History, focused on a life review and composing one’s life history using the guided autobiography method developed by Birren. The study used a crossover design with a one-week break interval between the two workshops. Each workshop was conducted weekly with a two-hour session over four weeks. Thirty-eight participants aged 60 to 80 attended both workshops between October and December 2021 at a community center in Tokyo, Japan. The sense of identity and integrity were assessed using the Japanese version of the Erikson Psychosocial Inventory Scale (EPSI) at baseline and after completing both workshops (9th week). The scores of both identity and integrity increased significantly among participants who completed two workshops in sequence. This crossover study showed no statistical difference in the effects of each workshop. Further work is needed to monitor the long-term impact of these workshops in clarifying one’s identity and achieving satisfaction with self in later life.

